# Preexisting *Trichinella spiralis* infection attenuates the severity of *Pseudomonas aeruginosa*-induced pneumonia

**DOI:** 10.1371/journal.pntd.0010395

**Published:** 2022-05-02

**Authors:** Shao Rong Long, Wen Xuan Shang, Miao Jiang, Jing Fei Li, Ruo Dan Liu, Zhong Quan Wang, Hualei Sun, Jing Cui

**Affiliations:** 1 Department of Parasitology, Medical College of Zhengzhou University, Zhengzhou, China; 2 Biology, School of Life Scence, Zhengzhou University, Zhengzhou, China; 3 Department of Nutrition, the First Affiliated Hospital of Zhengzhou University, Zhengzhou, China; NIH-National Institute for Research in Tuberculosis-ICER, INDIA

## Abstract

**Background:**

A range of helminth species involve the migration of developing larvae through the lung and establish chronic infections in the host that include potent immune regulatory effects. *Trichinella spiralis* is one of the most successful parasitic symbiotes. After released by intestinal female adult worms, newborn larvae of *T*. *spiralis* travel through the circulatory system to the lung and finally reach skeletal muscle cells. As unique inflammation modulator of intracellular parasitism, *T*. *spiralis* shows improved responses to autoimmune disease and viral pulmonary inflammation by exerting immunomodulatory effects on innate and adaptive immune cells.

**Methodology/Principal findings:**

C57BL/6 mice were divided into four groups: uninfected; helminth- *T*. *spiralis* infected; *P*. *aeruginosa* infected; and co-infected. Mice infected with *T*. *spiralis* were incubated for 6 weeks, followed by *P*. *aeruginosa* intranasal inoculation. Bronchial alveolar lavage fluid, blood and lung samples were analyzed. We found that *T*. *spiralis* induced Th2 response in the mouse lung tissue, increased lung CD4^+^ T cells, GATA3, IL-4, IL-5 and IL-13 expression. Pre-existing *T*. *spiralis* infection decreased lung neutrophil recruitment, inflammatory mediator IL-1β and IL-6 expression and chemokine CXCL1 and CXCL2 release during *P*. *aeruginosa-* pneumonia. Furthermore, *T*. *spiralis* co-infected mice exhibited significantly more eosinophils at 6 hours following *P*. *aeruginosa* infection, ameliorated pulmonary inflammation and improved survival in *P*. *aeruginosa* pneumonia.

**Conclusions:**

These findings indicate that a prior infection with *T*. *spiralis* ameliorates experimental pulmonary inflammation and improves survival in *P*. *aeruginosa* pneumonia through a Th2-type response with eosinophils.

## Author summary

Helminth infections elicit type 2 immunity, which influences host immune responses to additional threats, such as allergens, metabolic disease and other pathogens. *Pseudomonas aeruginosa* is one of the most common gram-negative pathogens causing pneumonia in immunocompromised patients. The mortality rate of ventilator associated pneumonia caused by *P*. *aeruginosa* is higher than that due to other pathogens. *Trichinella spiralis* is a zoonotic nematode of intracellular parasitism that infects a wide range of vertebrate hosts, including humans. There is a lung migratory phase in the life cycle of *T*. *spiralis*. In this study, we found that *T*. *spiralis* induced Th2 response in the mouse lung tissue. *T*. *spiralis* co-infected mice exhibited significantly more eosinophils and less neutrophils at 6 hours following *P*. *aeruginosa* infection, ameliorated pulmonary inflammation and improved survival in *P*. *aeruginosa* pneumonia. These findings suggest a pre-existing chronic helminth with a lung migration phase infection promotes the survival of bacterial airway co-infected host.

## Introduction

Pneumonia is the leading cause of childhood mortality worldwide, accounting for more than 1.6 million deaths per year [1]. *Pseudomonas aeruginosa* is one of the most common gram-negative pathogens causing pneumonia in immunocompromised patients [2]. The mortality rate of ventilator associated pneumonia caused by *P*. *aeruginosa* is higher than that caused by other pathogens [2]. During acute infection, *P*. *aeruginosa* disrupts the integrity of the lipid membrane causing epithelial cell damage and lung injury [3].

More than 1 billion people worldwide are infected with helminths [4]. Complex interactions between helminths and their host result in systemic effects on immunity, with a skewing towards type 2 (Th2) responses and profound consequences on the host immune milieu [5]. Helminths are one of the most common infectious agents of humans in developing countries, with children harboring a greater parasitic worm burden than adolescent and adults [6,7]. While helminth-driven Th2 immunity can be effective at expelling parasites, numerous studies now highlight the effects this may have on human infectious, inflammatory and metabolic diseases [8–12]. Given chronic helminth infection and acute infectious pneumonia likely overlap among populations of the world and the complex influence that helminth infection can exert on host immunity, a helminth and bacterium coinfection was developed in our previous study. We found that a gut-restricted *Heligmosomoides polygyrus* infection in mice influenced the early innate immune response within the lung challenged with the respiratory pathogen *P*. *aeruginosa*, co-infected mice exhibited significantly more airspace neutrophil infiltration at 6 hours following *P*. *aeruginosa* infection and exhibited an improved rate of survival compared with bacterial infected alone [13].

*H*. *polygyrus* is a strictly murine enteric helminth, whose life cycle does not involve direct contact with the lung. It’s worth noting that the life cycles of a range of helminth species involve the migration of developing larvae through the lung of the infected host. Immune responses are heavily influenced by helminths passing through the lung or through releasing their immunomodulators [14]. In the case of the helminth *Trichinella spiralis*, the larvae of which travel through the circulatory system to the lung and finally reach skeletal muscle cells [15]. It is known as a zoonotic nematode of intracellular parasitism that infects a wide range of vertebrate hosts, including humans. Multiple studies have confirmed the anti-inflammatory effect of *T*. *spiralis* in animal models, such as allergies, autoimmune disease, rheumatoid arthritis and viral pulmonary inflammation [16,17]. Here, we investigated the role of *T*. *spiralis* infection in modulating respiratory pathogen-*P*. *aeruginosa* infection. This study sought to determine if a chronic infection *by T*. *spiralis*, the parasite with pulmonary migration in larval stage, could influence the severity of bacterial pneumonia in mice and its possible mechanism.

## Material and methods

### Ethics statement

All experiment procedures involving animals were carried out on the basis of the protocol authorized by the Institutional Life Science Ethics Committee, Zhengzhou University, China (Permission No. SCXK 2017–0001).

### Bacterial strain

All bacterial infections were carried out with *P*. *aeruginosa* strain PA14. Bacterial cultures were grown aerobically in Luria Bertani broth over nightat 37°C, 200 RPM. Prior to infection, 1 mL of overnight culture was pelleted (10,000 rpm, 15 minutes, 4°C), washed 2 times with 1 mL of phosphate buffered saline (PBS), and re-suspended in 2 mL of PBS. The optical density for the strain was obtained at 600 nm using the Spectrumlab 722SP (LengGuang 7ech, China). The stock solution was used to make the appropriate dilutions.

### Mice

Pathogen-free, 8-week-old female C57BL/6 mice were purchased from the Experimental Animal Center of Henan Province. They were fed autoclaved food and water. The animal care and experiment were performed according to the guidelines for the Care and Use of Laboratory Animals of the Ministry of Science and Technology of P. R. China (2006).

### Helminth infection

The helminth parasite *T*. *spiralis* (ISS534) was collected as previously described [18]. C57BL/6 mice were randomly separated into two groups; the helminth infection group was administered 100 muscle larvae orally by gavage with a 21-gauge feeding needle (BOLIVIAN DOVE, Shanghai). *T*. *spiralis* infection was incubated for 42 days prior to infection of the mice with *P*. *aeruginosa*.

### Intranasal bacterial inoculation

After 42 days of helminth infection, mice were anesthetized by intraperitoneal administration of a freshly prepared mixture of tribromoethanol solution (25mg/ml, Sigma). Tribromoethanol was dissolved in 2-methyl-2-butanol (1g/mL, Sigma) and was subsequently diluted to working concentrations in nano-pure H_2_O. A random subset of mice from both the naïve group and *T*. *spiralis*- infected group were infected with *P*. *aeruginosa* through intranasal inoculation (30 μl, 5×10^5^ CFU/mouse). The groups that were not infected with bacteria were inoculated intranasally with the same volume of sterile PBS as mice receiving PA14 infection. After intranasal inoculation, the mice were placed in the cage and monitored for 6 hours or 7 days, at which time the mice were euthanized and samples were obtained and processed.

### Bronchial alveolar lavage fluid harvest

Bronchial alveolar lavage fluid (BALF) samples were obtained and processed as previously described [13]. Lungs were flushed with 2 mL of PBS supplemented with 0.3% FBS and 300 μM EDTA. Cell count and viability measurements using trypan blue stain were performed. Cells were blocked with anti-CD16/CD32 (BD Biosciences) for 20 min on ice and processed for flow cytometry.

### Bacterial recovery from mouse lungs

Whole lung tissue, which BALF was not acquired from, was removed surgically and placed into 2 mL tubes which contained 3 3 mm metal beads with 1 mL of PBS and stored on ice. Tubes were placed in the KZ-III-F (Servicebio, 70 Hz, homogenization time: 70 sec). Lung homogenate were plated on *Pseudomonas* isolation agar plates and grown at 37°C overnight. Colonies were counted, and the total CFU/mL recovered from the homogenized tissue samples was calculated.

### Lung histology and immunohistochemistry assay

At necropsy, left lung tissues were collected, formalin-fixed and embedded in paraffin. The processed tissues were sectioned into 5μm thick slices. For inflammation and injury assessment, hematoxylin and eosin (H&E) staining was performed. For eosinophils detection, EPX (Eosinophil peroxidase heavy chain, Bioss) immunohistochemistry staining according to manufacturer’s instruction was performed to distinguish eosinophils in lung parenchyma. The staining on slides was visualized using Olympus Bx43F orthotopic microscope. The inflammatory score was calculated as following features: lung interstitial edema, haemorrhage, and neutrophil infiltration [19] **(Table A in S1 Text)**. To quantify the infiltrating cells or eosinophils, the acquired images from the microscope were processed using the ImageJ software. The quantification was performed by counting at least 10 random fields of view with 40x objective and calculating the average and standard deviation.

### Flow cytometry preparation and analysis

The lung tissues were first cut into small pieces and incubated with 1 mg/mL collagenase D (Sigma) and 1 mg/mL DNAse I (Solarbio) at 37°C for 30 mins. Collagenase was inactivated with 1 mL sterile DMEM containing 10% FBS; the digested tissues were transferred to a 70-μM nylon cell strainer and disrupted using a syringe plunger to obtain single cell suspensions. Following lung tissue processing, the resulting single cell suspension, peripheral blood and BALF samples were processed in 1mL Red Blood Cell Lysing Bufer (Sigma). The cellular populations from different groups were identified through fluorescence conjugated antibodies. Stained samples were analyzed with BD FACSCanto. Fluorescence conjugated antibodies were listed as **Table B in S1 Text**. The corresponding isotype control antibodies were purchased from Biolegend and used in the staining of flow cytometry analysis. Dead cells and debris were excluded from analysis via size exclusion.

### RNA isolation and qRT-PCR

Total RNA was prepared from lung tissues using TRIzol reagent (Takara) and reverse transcribed into cDNA using PrimeScript RT reagent kit (TaKaRa) following the manufacturer’s recommendations. The cDNA samples were then tested for the expression of IL-1β, IL-5, IL-6, IL-17, IFN-γ, GATA3, IL-4, IL-13, ZO-1, claudin-5, VE-cadherin, CXCL1 and CXCL2 by real-time quantitative PCR using TB Green Premix Ex Taq (TaKaRa) on a 7500 Fast Real-Time PCR system (Applied Biosystems). GAPDH was used as the housekeeping control. The gene expression level was normalized by subtracting the expression level of GAPDH of the same group, and the different expression levels were calculated using the comparative Ct (2^−ΔΔCt^) method.

### Statistical analysis

All results were expressed as the mean ± SD. For normally distributed data, one-way ANOVA test was used to compare three or more groups and Student’s t test to compare two groups. Bonferroni Correction was used to adjust multiple comparisons. A Kaplan-Meier analysis followed by a log rank (Mantel-Cox) test was used to compare the survival between the two groups. A *P* value of < 0.05 was considered statistically significant.

## Results

### *T*. *spiralis* coinfection ameliorates pulmonary inflammation and improves survival in *P*. *aeruginosa* pneumonia

**Fig 1:**
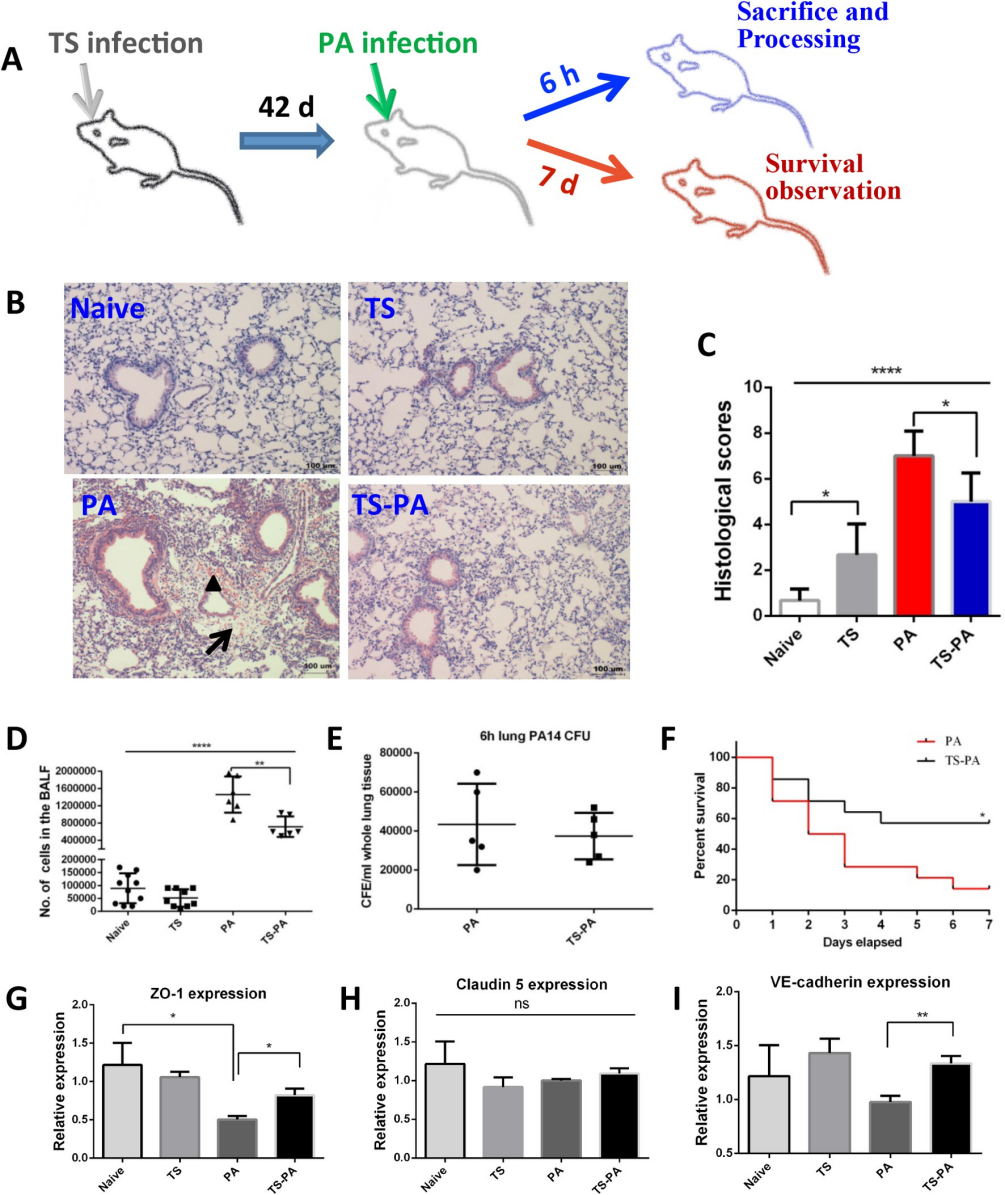
*T*. *spiralis* coinfection ameliorates pulmonary inflammation and injury in *P*. *aeruginosa* pneumonia. **(A)** Schematic of the *T*. *spiralis*- *P*. *aeruginosa* coinfection experimental process. **(B)** Lung histological examination 6 h post *P*. *aeruginosa* infection, arrow: perivascular exudates, triangle: haemorrhage. **(C)** Histological scores were from 3 mice per group, 2 lobes examined per mouse. **(D)** The result of BALF cell count, results of 2 independent experiments were combined. **(E)** *P*. *aeruginosa* CFU in the whole lung tissue without BALF acquisition, results of 2 independent experiments were combined. **(F)** Coinfection with *T*. *spiralis* increased mouse survival, data shown was a compilation of 3 independent experiments, with a total of 14 mice cumulatively in each of the 2 groups. **(G-H)** mRNA expression of zonula occludens 1 (ZO-1) **(G)**, claudin-5 **(H)** and vascular endothelial cadherin (VE-cadherin) **(I)** was evaluated in lung samples by RT-qPCR; values are the relative expression compared to the naïve mice, data showed are from one of three experiments performed showing similar results. Naïve: uninfected; TS: *T*. *spiralis* infected; PA: *P*. *aeruginosa* infected; TS-PA: *T*. *spiralis* and *P*. *aeruginosa* co-infected. Data are shown as mean±SD; *P< 0.05; **P< 0.01; ****P<0.0001; ns: no significant difference.

A coinfection model was developed to evaluate whether *T*. *spiralis* infection can influence the response to infection with *P*. *aeruginosa* of mice. After 42 days of *T*. *spiralis* infection, a random subset of mice from both the naïve group and *T*. *spiralis* -infected group were infected with *P*. *aeruginosa* through intranasal inoculation; mice were placed in the cage for 6 hours or 7days and then sacrificed **(Fig 1A)**. To assess the impact of *T*. *spiralis* infection on pulmonary inflammation, detailed lung histological examination was performed. After 6 h *P*. *aeruginosa* infection, marked differences in lung injury were observed between *T*. *spiralis* coinfection and *P*. *aeruginosa* infection alone mice. As shown in **Fig 1B**, perivascular exudates, haemorrhage, and cell infiltration were exhibited in *P*. *aeruginosa* infection mice, but were noticeably attenuated in *T*. *spiralis* coinfection mice. *T*. *spiralis* coinfection mice had less severe histological scores, based on hemorrhage, interstitial edema, and neutrophil infiltration (**Fig 1C**). To examine the inflammatory cell infiltration into the lung airspace during the process of initiation of *P*. *aeruginosa* pneumonia, BALF was collected. The total number of cells within BALF was significantly increased following 6 h *P*. *aeruginosa* infection (**Fig 1D**). Additionally, the total number of cells was decreased significantly further in BALF collected from mice co-infected with *T*. *spiralis* and *P*. *aeruginosa*, compared to that detected in mice infected with *P*. *aeruginosa* alone (**Fig 1D**). Despite the significant difference in cellular infiltration of the airspace between bacterial infected and co-infected mice, no significant diferences in bacterial burden were observed over a 6-hour bacterial infection period between *P*. *aeruginosa* infected alone and co-infected mice (**Fig 1E**).

Increased pulmonary permeability is a hallmark of acute lung injury. Basal endothelial cell barrier control is mediated by cell junctions containing adherens junction (AJ) and tight junction (TJ) protein complexes [20]. To investigate the effects of *T*. *spiralis* coinfection on pulmonary vascular permeability, at 6 hours post bacterial infection, the levels of expression of TJs (ZO-1, claudin-5) and AJ (VE-cadherin) mRNA were evaluated by RT-qPCR in lung tissue. The primers used for RT-qPCR are listed in **Table 1**. Significant reduction of ZO-1 mRNA expression occurred in *P*. *aeruginosa*- infected mice only (**Fig 1G**), when compared to the naïve mice. However, during *P*. *aeruginosa* infection of the lung, the decreased expression of ZO-1 and VE-cadherin was significantly reversed by pretreatment with *T*. *spiralis* (**Fig 1G and 1I**). The claudin-5 mRNA expression in lung was not affected by *T*. *spiralis* or *P*. *aeruginosa* infection (**Fig 1H**).

**Table 1:** Primers used for qPCR analysis.

Genes	Primer	Sequence (5′→3′)
IL-1β	Forward	AGCTCTCCACCTCAATGGAC
	Reverse	AGGCCACAGGTATTTTGTCG
IL-5	Forward	TGACAAGCAATGAGACGATGAGG
	Reverse	ACCCCCACGGACAGTTTGATTC
IL-6	Forward	TAGTCCTTCCTACCCCAATTTCC
	Reverse	TTGGTCCTTAGCCACTCCTTC
IL-17	Forward	CCACGTCACCCTGGACTCTC
	Reverse	CTCCGCATTGACACAGCG
IFN-γ	Forward	GGAACTGGCAAAAGGATGGTGAC
	Reverse	GCTGGACCTGTGGGTTGTTGAC
CXCL1	Forward	GACTCCAGCCACACTCCAAC
	Reverse	TGACAGCGCAGCTCATTG
CXCL2	Forward	GAAGTCATAGCCACTCTCAAGG
	Reverse	CCTCCTTTCCAGGTCAGTTAGC
ZO-1	Forward	TGAACGCTCTCATAAGCTTCGTAA
	Reverse	ACCGTACCAACCATCATTCATTG
claudin-5	Forward	TCTGCTGGTTCGCCAACAT
	Reverse	CGGCACCGTCGGATCA
VE-cadherin	Forward	GCGCAGCATCGGGTACTC
	Reverse	GCTTGGTTATTCGGAAGAATTGG
IL-4	Forward	GGTCTCAACCCCCAGCTAGT
	Reverse	GCCGATGATCTCTCTCAAGTGAT
IL-13	Forward	CCTGGCTCTTGCTTGCCT
	Reverse	GGTCTTGTGTGATGTTGCTCA
GATA3	Forward	CTCGGCCATTCGTACATGGAA
	Reverse	GGATACCTCTGCACCGTAGC
GAPDH	Forward	GGTTGTCTCCTGCGACTTCA
	Reverse	TGGTCCAGGGTTTCTTACTCC

To further investigate the impact of *T*. *spiralis* coinfection on the course of the bacterial infection, we examined animal survival at day 7 post bacterial infection. After 6 weeks of *T*. *spiralis* infection, the mice were infected with *P*. *aeruginosa* through intranasal inoculation, mice were placed in the cage and monitored for 7 days, Results showed that *T*. *spiralis* coinfection significantly improved survival compared to *P*. *aeruginosa* infection alone (**Fig 1F**). Taken together, these findings indicate *T*. *spiralis* coinfection ameliorate *P*. *aeruginosa* pneumonia.

### *T*. *spiralis* coinfection results in decreased neutrophil recruitment in the lung airspace and parenchyma

**Fig 2:**
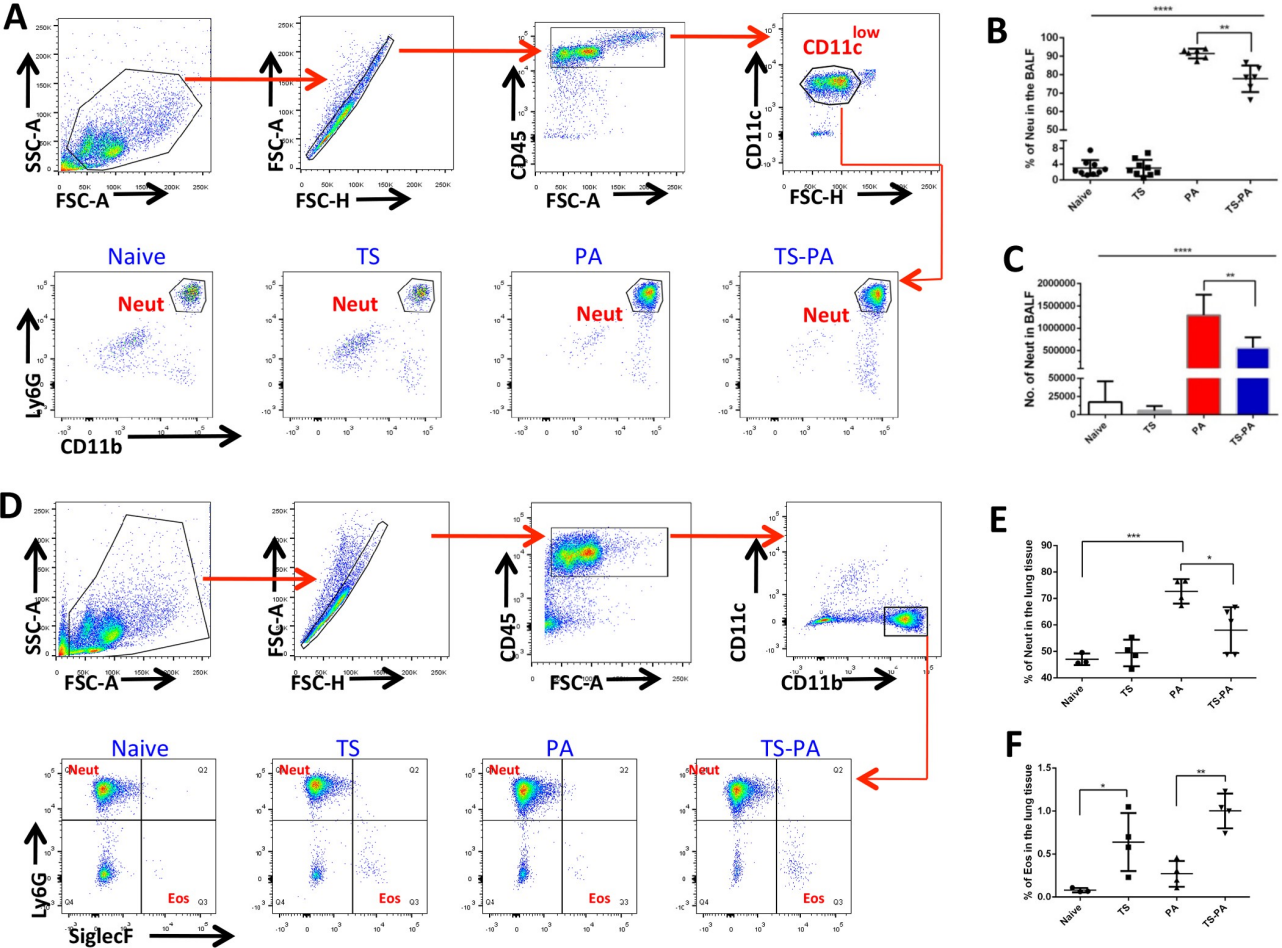
Coinfection with *T*. *spiralis* decreases neutrophil recruitment in the *P*. *aeruginosa* infected airspace and parenchyma of the lung. **(A)** Gating strategy of BALF neutrophils (CD45^+^CD11c^low^Ly6G^+^CD11b^+^) in flow cytometry analysis (FACS). The percentage **(B)** and absolute number **(C)** of neutrophils in the BALF were compared between control and *T*. *spiralis* infected mice in the presence and absence of *P*. *aeruginosa* infection; results of 2 independent experiments were combined. **(D)** Gating strategy of lung parenchyma neutrophils (CD45^+^CD11b^+^CD11c^-^Ly6G^+^SiglecF^-^) and eosinophils (CD45^+^CD11b^+^CD11c^-^Ly6G^-^SiglecF^+^) in FACS. The percentage of neutrophils **(E)** and eosinophils **(F)** in the lung parenchyma was compared among groups; results were from one of three experiments performed showing similar results. Naïve: uninfected; TS: *T*. *spiralis* infected; PA: *P*. *aeruginosa* infected; TS-PA: *T*. *spiralis* and *P*. *aeruginosa* co-infected; Neut: neutrophils; Eos: eosinophils. Data are shown as mean±SD. *P< 0.05;**P< 0.01, ***P< 0.001; ****P< 0.0001.

The severe neutrophilic inflammation is fatal during acute lung injury. To evaluate the role of *T*. *spiralis* infection in the modulation of initial responses to bacterial infection, we exposed the airway of *T*. *spiralis*- infected and control mice to *P*. *aeruginosa* and examined neutrophil recruitment to the lung at 6 hours post infection. The neutrophil in the BALF were identified as CD45^+^CD11c^low^Ly6G^+^CD11b^+^ (**Fig 2A**), infection of the lung with *P*. *aeruginosa* resulted in statistically significant increase in the percentage (**Fig 2B**) and absolute quantities (**Fig 2C**) of neutrophil infiltrating the airspace in both the absence and presence of *T*. *spiralis* infection when compared to their respective controls; naïve or *T*. *spiralis* infection alone. Meanwhile, there was a statistically significant decrease in the quantity of neutrophils that infiltrated the airspace of co-infected mice when compared to *P*. *aeruginosa* infection alone (**Fig 2B and 2C**). Our analysis of lung tissues with BALF acquisition revealed that the percentage of CD45^+^CD11b^+^CD11c^-^Ly6G^+^SiglecF^-^ neutrophils (**Fig 2D**) significantly decrease in *T*. *spiralis* co-infected hosts compared to *P*. *aeruginosa*-infected alone hosts (**Fig 2E**). The neutrophil in lung parenchyma increased in the hosts infected with *P*. *aeruginosa* when compared to naïve control mice, while there was no significant difference in the percentage of lung parenchyma neutrophil between co-infected and *T*. *spiralis* infected alone mice (**Fig 2E**).

### *T*. *spiralis* coinfection changes the expression of inflammatory mediators during *P*. *aeruginosa* pneumonia

**Fig 3:**
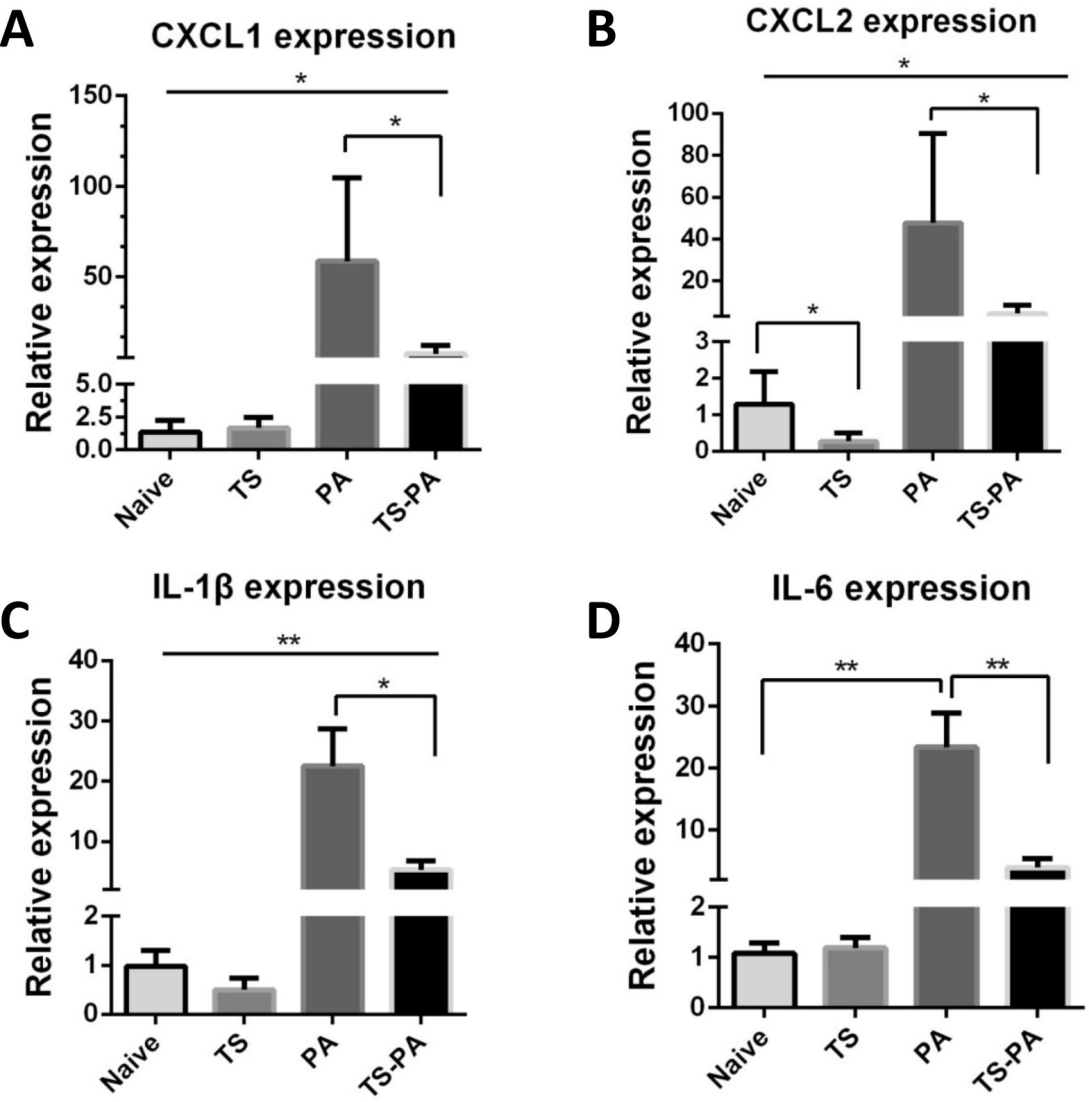
Prior *T*. *spiralis* infection has impact on inflammatory mediators. **(A–D)** Lung tissues were collected from naïve, *T*. *spiralis*-infected, *P*. *aeruginosa*-infected, and *T*. *spiralis* -co-infected mice. Total RNA was isolated from the lung tissues. CXCL1, CXCL2, IL-6 and IL1-β expression was determined using RT-qPCR. Values are the relative expression compared to the naïve mice. The data shown are means±SD from one of three experiments performed showing similar results. Naïve: uninfected; TS: *T*. *spiralis* infected; PA: *P*. *aeruginosa* infected; TS-PA: *T*. *spiralis* and *P*. *aeruginosa* co-infected. *P< 0.05; **P< 0.01.

The presence of *T*. *spiralis* infection during *P*. *aeruginosa* airway infection resulted in reduced neutrophil migration to the infected lung (**Fig 2**). Inflammatory cytokines and chemokines play an important role in the neutrophil recruitment. Expression of CXCL1, CXCL2, IL-6, and IL-1β were evaluated by RT-qPCR in lung tissue. The primers used for RT-qPCR are listed in **Table 1**. During *P*. *aeruginosa* infection of the lung, while expression levels of CXCL1, CXCL2 and IL-1β were increased in the presence or absence of a concomitant helminth *T*. *spiralis* infection, *T*. *spiralis* coinfection had a less expression when compared to *P*. *aeruginosa* infection alone **Fig 3A–3C**). Besides, *T*. *spiralis* infection reduced the CXCL2 lung expression in the absence of *P*. *aeruginosa* infection (**Fig 3B**). IL-6 lung expression was also increased in the *P*. *aeruginosa* infection when compared to the naïve mice, however, IL-6 expression level was significantly suppressed in the presence of *T*. *spiralis* coinfection when compared to *P*. *aeruginosa*- infected alone mice **(Fig 3D)**.

### *T*. *spiralis* coinfection results in increasing eosinophil recruitment into the lung during *P*. *aeruginosa* pneumonia

**Fig 4:**
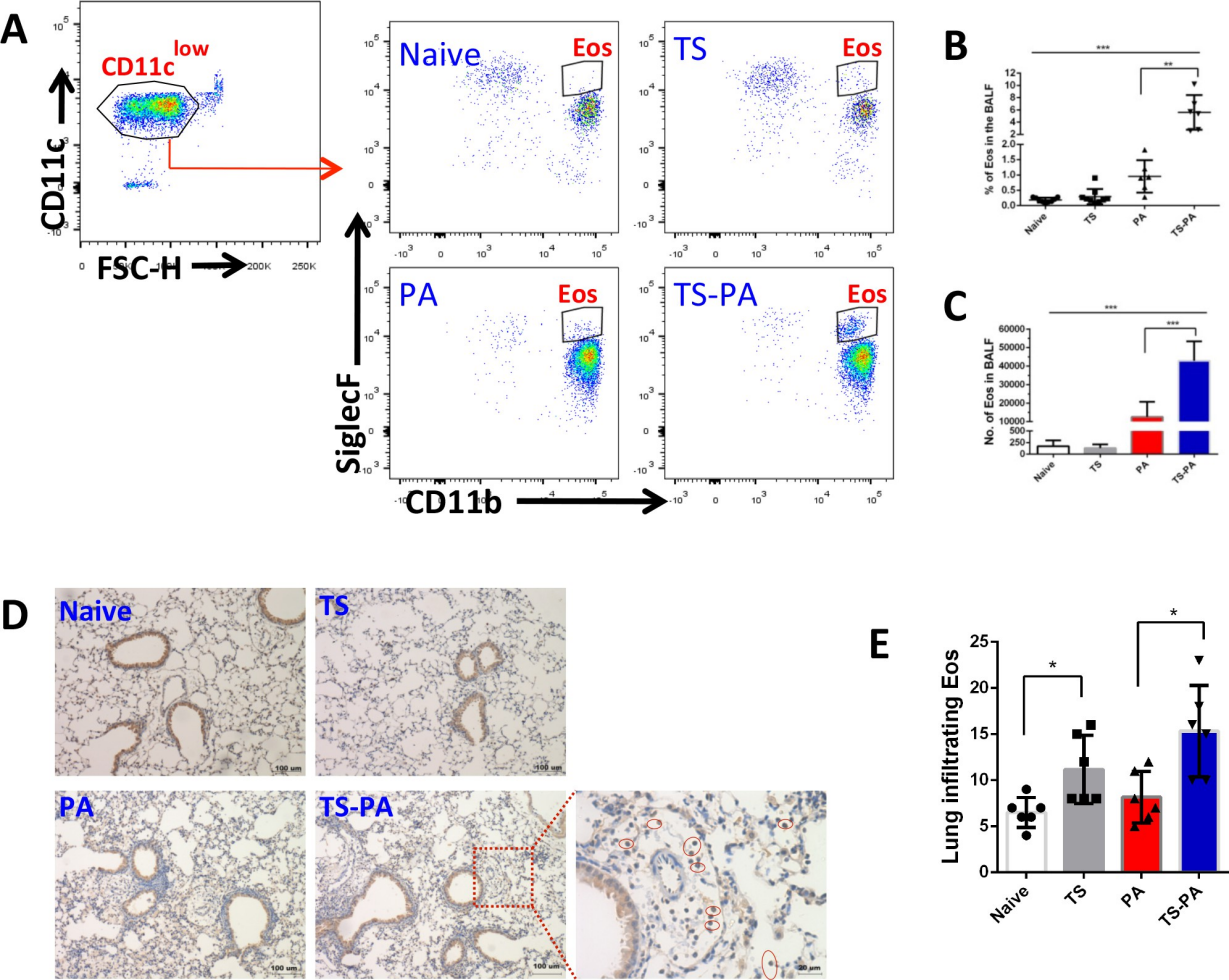
Coinfection with *T*. *spiralis* increases the lung eosinophils during *P*. *aeruginosa* pneumonia. **(A)** Gating strategy of BALF eosinophils (CD45^+^CD11c^low^SiglecF^+^CD11b^+^) in flow cytometry analysis. The percentage **(B)** and absolute number **(C)** of eosinophil in the BALF were compared between control and *T*. *spiralis* infected mice in the presence and absence of *P*. *aeruginosa* infection. Rresults of 2 independent experiments were combined. **(D)** Representative images and **(E)** semi-quantification of EPX immunohistochemistry staining (circle) in mouse lung tissue. In semi-quantification, each point was the average score of 10 random fields of each mouse. Data are shown as mean±SD. Naïve: uninfected; TS: *T*. *spiralis* infected; PA: *P*. *aeruginosa* infected; TS-PA: *T*. *spiralis* and *P*. *aeruginosa* co-infected; Eos: eosinophils. *P< 0.05;**P< 0.01; ***P< 0.001.

Increase in eosinophils is a classical feature of helminth infections, to assess the possible function of eosinophils in the process of *P*. *aeruginosa* pneumonia; we examined eosinophils in the mice with/without infection. Eosinophils in the BALF were identified as CD45^+^CD11c^low^SiglecF^+^CD11b^+^ (**Figs 2A and 4A**). Infection of the lung with *P*. *aeruginosa* increased the percentage (**Fig 4B**) and absolute quantities (**Fig 4C**) of eosinophils in the airspace in both the absence and presence of *T*. *spiralis* infection when compared to their respective controls; naïve or *T*. *spiralis* infection alone. And there was a statistically significant increased eosinophils in the airspace of *T*. *spiralis* co-infected mice when compared to *P*. *aeruginosa* infection alone mice (**Fig 4B and 4C**). A similar trend was observed regarding the percent of CD45^+^CD11b^+^CD11c^-^Ly6G^-^SiglecF^+^ eosinophils derived from lung parenchyma **(Fig 2D)**, *T*. *spiralis* infection increased the frequency of eosinophils within the lung parenchyma in both the absence and presence of *P*. *aeruginosa* infection when compared to their respective controls, naïve or *P*. *aeruginosa* infection alone (**Fig 2F**). Similarly, immunohistochemistry staining **(Fig 4D)** suggested that the number of eosinophils in the lung parenchyma was significantly increased in the *T*. *spiralis* infection mice when compared to their respective controls, naïve or *P*. *aeruginosa* infection alone mice **(Fig 4E).** In addition, *T*. *spiralis* infection increased the frequency of eosinophil within the peripheral blood in both the absence and presence of *P*. *aeruginosa* infection when compared to their respective controls **(S1 Fig)**.

### *T*. *spiralis* infection induces Th2-type polarization in the lung

**Fig 5:**
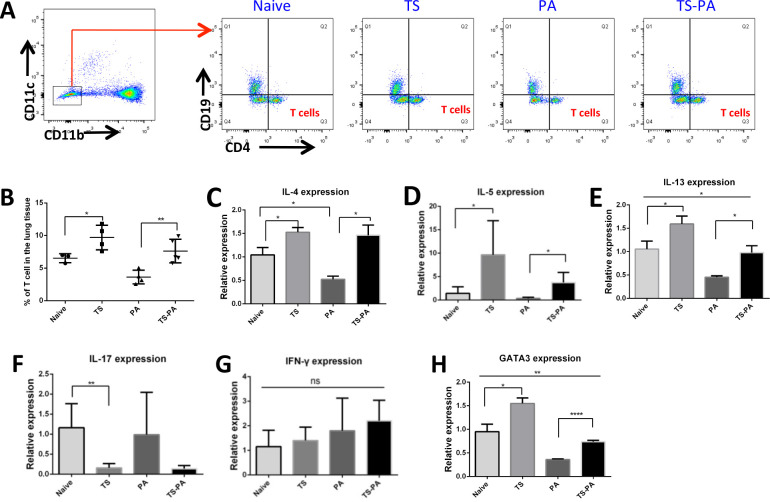
T. *spiralis* infection increases Th2 cells population in the lung tissues. **(A)** FACS analysis to isolate the CD4^+^ T-cell population in the lung tissue. Gating strategy of lung tissue CD4^+^-T cells (CD45^+^CD11b^-^CD11c^-^CD4^+^CD19^-^) in flow cytometry analysis. **(B)** The percentage of CD4^+^ T cells in the lung tissue was compared between control and *T*. *spiralis* infected mice in the presence and absence of *P*. *aeruginosa* infection. **(C-H)** Lung tissues were collected from naïve, *T*. *spiralis*-infected, *P*. *aeruginosa*-infected, and *T*. *spiralis* -co-infected mice. Total RNA was isolated from lung tissues. IL-4 **(C)**, IL-5 **(D)**, IL-13 **(E)**, IL-17 **(F)**, IFN-γ **(G)** and GATA3 **(H)** expression were determined using RT-qPCR. Values are the relative expression compared to the naïve mice. Naïve: uninfected; TS: *T*. *spiralis* infected; PA: *P*. *aeruginosa* infected; TS-PA: *T*. *spiralis* and *P*. *aeruginosa* co-infected; Eos: eosinophils. The data shown are means±SD from one of three experiments performed showing similar results. *P<0.05; **P<0.01; ****P<0.0001; ns: no significant difference.

CD4^+^T cells were identified as CD45^+^CD11b^-^CD11c^-^CD4^+^CD19^-^ (**Figs 2D and 5A**). The data showed that *T*. *spiralis* infection elicited higher quantities of CD4^+^ T-cells in the lung tissues when compared to naïve mice irrespective of bacterial infection **(Fig 5B).** To determine the type of the CD4^+^T cells, at 6 hours post bacterial infection, gene expression levels of Th1 cytokine IFN-γ, Th2 cytokines IL-4, IL-5, IL-13 and Th17 cytokine IL-17 in the lung was examined. The primers used for RT-qPCR are listed in **Table 1**. During *P*. *aeruginosa* infection of the lung, expression levels of IL-4 and IL-13 were decreased in the absence of a concomitant helminth *T*. *spiralis* infection (**Fig 5C and 5E).** *T*. *spiralis* infection were associated with a significant upregulation of IL-4, IL-5 and IL-13 expression in lung tissues in both the absence and presence of *P*. *aeruginosa* infection when compared to their respective controls, naïve or *P*. *aeruginosa* infection alone (**Fig 5C–5E**). In addition, *T*. *spiralis* infection reduced the lung IL-17 expression when compared to the naïve mice (**Fig 5F**). The lung IFN-γ expression did not appear alter in any context (**Fig 5G**). Besides, *T*. *spiralis* infection were also associated with a significant upregulation of GATA3 transcription factor expression in lung tissues in both the absence and presence of *P*. *aeruginosa* infection when compared to their respective controls, naïve or *P*. *aeruginosa* infection alone (**Fig 5H**); *P*. *aeruginosa* infection down-regulated the expression level of GATA3 in the presence or absence of a concomitant *T*. *spiralis* infection. These results demonstrate that *T*. *spiralis* infection promotes the development of Th2 immune response in lung.

## Discussion

Helminth infections are known to be powerful modulators of the human immune response, and numerous studies now highlight that it may have effects on human infectious, inflammatory, and metabolic diseases [21]. The helminth *Trichinella spiralis* is the causative agent of trichinosis. Humans contract *T*. *spiralis* by consuming uncooked meat that carries encysted *T*. *spiralis* larvae in muscle tissue; then larvae are released by gastric fluids, molt and mature into adult worm. The female adult worms release newborn larvae after copulation, which travel through the circulatory system to reach skeletal muscle cells [16]. *T*. *spiralis* is one of the most successful parasitic symbiotes, ensuring its survival and immune dialogue with the host. Multiple studies have confirmed the anti-inflammatory effect of *T*. *spiralis* in animal models. Mice with a persistent *T*. *spiralis*-mediated type 2 immunity redirects the mucosal immune system from a Th1 to a protective Th2 response with a reduction in the severity of IBD [22]. Chronic infection with *T*. *spiralis* can mediate protection against allergic asthma by eliciting a regulatory T cell population and increasing IL-10 levels [23]. Evidence also indicates that *T*. *spiralis* infection has attenuated influenza or respiratory syncytial virus -associated pathologies in mice [17,24]. Yet, not a single study has reported the effect of *T*. *spiralis* infection in regulating bacterial infection- induced pneumonia. Here, we addressed this question by co-infecting mice with *T*. *spiralis* and respiratory pathogen–*P*. *aeruginosa*. Findings demonstrated that a prior infection with *T*. *spiralis* could ameliorate experimental pulmonary inflammation and improved survival in *P*. *aeruginosa* pneumonia through a Th2-type response with eosinophils.

An in vivo coinfection model system involving infection with *T*. *spiralis*, followed by airway administration of a lung bacterial pathogen (*P*. *aeruginosa*), was developedand characterized to analyze the effects of helminth parasites with pulmonary migration infection on the bacterial infection of the airway. Consistent with our previous report [13], mice infection with helminth had an enhanced Th2 response in the lung tissue; the GATA3 transcription factor, type2 cytokines-IL-4, IL-5, IL-13 and CD4^+^ T cells increased in the mice infection with *T*. *spiralis*. In the previous study, we observed that concurrent intestinal helminth- *H*. *polygyrus* infections increased the airspace neutrophil recruitment at the early stages of bacterial lung infection [13]. However, FACS analysis results of present study showed that *T*. *spiralis* coinfection resulted in depressed neutrophil transepithelial migration into the airspace and lung tissue. Chen and colleagues had demonstrated that *Nippostrongylus brasiliensis*, a helminth with an obligatory lung migratory phase, suppressed the neutrophil infiltration into the lungs upon infection through Th2 cytokine receptor signaling [25]. The diverse outcomes of neutrophil infiltration into the lung during *P*. *aeruginosa* infection may be due to differences in the life cycle of the two parasites: *H*. *polygyrus* is a strictly gut-restricted helminth, while *T*. *spiralis* with a lung migratory phase. Neutrophils released from the bone marrow are regulated by the signaling of CXC chemokine receptors (CXCR) 2 and 4 [26]. CXCR2 can bind to human CXCL8 and mouse CXCL1 and CXCL2, which can promote the mobilization of neutrophils and their entry into the bloodstream. [27,28]. In the present study, the observation of depressed neutrophil transepithelial migration was further supported by the decreased level of chemokines-CXCL1 and CXCL2 in the lung tissue of *T*. *spiralis* co-infected mice. And also *T*. *spiralis* coinfection reduced the inflammation cytokines of IL-1β and IL-6 in the mouse lung tissue.

Eosinophils are circulating and tissue-resident leukocytes that have potent proinflammatory effects in a number of diseases, such as asthma, eosinophilic granulomatosis with polyangiitis and eosinophilic chronic rhinosinusitis [29]. In addition to their proinflammatory effects, growing evidence has demonstrated eosinophils have regulatory effects on various immune responses [30]. Researchers find eosinophils exert a protective effect in experimental colitis via production of anti-inflammatory lipid mediators or modulation of harmful type 1 responses [31,32], protecting the host against virus via production of oxidant agents, extracellular traps, and release of antiviral type I cytokines [33]. Eosinophils are also known as the terminal effector cells during helminth infection [30]; the most potent activator of which is the type 2 cell-produced cytokine- IL-5 [29]. In our study, helminth *T*. *spiralis* infection upregulated IL-5 expression in lung tissues. Our data also revealed that eosinophil in the lung parenchyma and peripheral blood increased in *T*. *spiralis* infected mice; moreover, *T*. *spiralis* coinfection increased eosinophils transepithelial migration into the lung airspace during *P*. *aeruginosa* infection. Clinical investigations showed that the acute lung injury survivors exhibited an increased number of eosinophils in the lung [34] and peripheral blood [35] compared to the non-survivors. Consistent with these reports, our results showed that *T*. *spiralis* coinfection improved lung endothelial cell permeability and survival during *P*. *aeruginosa* pneumonia, and eosinophils in *T*. *spiralis* co-infected mice was increased; suggesting a protective role of eosinophils in acute lung bacterial infection. Whether eosinophil is responsible for ameliorating pulmonary inflammation of co-infected mice is an important question that remains to be determined. Parasites are in some ways harmful to the host; treatment with live worms is not a very attractive notion, the excretion–secretion products released by *Trichinella* or parasite-derived proteins may be used as alternatives in the future study.

In conclusion, our data indicate that a prior infection with *T*. *spiralis* limits the development of *P*. *aeruginosa* pneumonia by decreasing neutrophil recruitment and inducing a Th2-type response with eosinophils. These findings suggest a pre-existing chronic helminth with a lung migration phase infection promotes the survival of bacterial airway co-infected host.

## Supporting information

S1 Text Table A in S1 Text. Assessment of histopathological scores. **Table B in S1 Text.** Fluorescent antibodies used in the Flow cytometry.
(DOC)

S1 Fig **(A)** Gating strategy of peripheral blood eosinophils (CD45^+^SiglecF^+^CD11b^+^) in flow cytometry analysis. **(B)** The percentage of eosinophils in the peripheral blood was compared among groups. Naïve: uninfected; TS: *T*. *spiralis* infected; PA: *P*. *aeruginosa* infected; TS-PA: *T*. *spiralis* and *P*. *aeruginosa* coinfected; Eos: eosinophils. The data shown are means±SD from one of three experiments performed showing similar results. *P<0.05; **P<0.01; ***P<0.001.
(TIF)
