# Supplementary material for: Preexisting Trichinella spiralis infection attenuates the severity of Pseudomonas aeruginosa-induced pneumonia
Source: PLoS Negl Trop Dis. 2022 May 2;16(5):e0010395. doi: 10.1371/journal.pntd.0010395 (PMC9098000; doi:10.1371/journal.pntd.0010395)
Supplement: S1 Text — Assessment of histopathological scores. Table B in S1 Text. Fluorescent antibodies used in the Flow cytometry. (DOC) [file pntd.0010395.s001.doc]

**Supplementary Information**

**Table A in S1 Text. Assessment of histopathological scores.**

| **lung interstitial edema** | **haemorrhage** | **neutrophil infiltration** | **Score** |
| --- | --- | --- | --- |
| no injury | no injury | no injury | 0 |
| limited injury | limited injury | limited injury | 1 |
| visible injury | visible injury | visible injury | 2 |
| severe injury | severe injury | severe injury | 3 |

**Table B in S1 Text.** Fluorescent antibodies used in the Flow cytometry.

| **Target** | **Fluorescent** | **Manufacturer** | **Clone** | **Cat. No.** |
| --- | --- | --- | --- | --- |
| CD45 | PE/Cyanine7 | Biolegend | 30-F11 | 103114 |
| CD4 | APC/Cyanine7 | Biolegend | GK1.5 | 100414 |
| CD19 | eFluor 506 | eBiosciience | eBio103 | 2107357 |
| CD11c | Alexa Fluor 700 | eBiosciience | N418 | 2133304 |
| CD11b | PerCP-Cyanine5.5 | eBiosciience | M1/70 | 2102846 |
| Ly6G | Alexa Fluor 488 | Biolegend | 1A8 | 127626 |
| SiglecF | PE | Biolegend | S17007L | 155506 |
