# Supplementary figures and images for: Preexisting Trichinella spiralis infection attenuates the severity of Pseudomonas aeruginosa-induced pneumonia
Source: PLoS Negl Trop Dis. 2022 May 2;16(5):e0010395. doi: 10.1371/journal.pntd.0010395 (PMC9098000; doi:10.1371/journal.pntd.0010395)

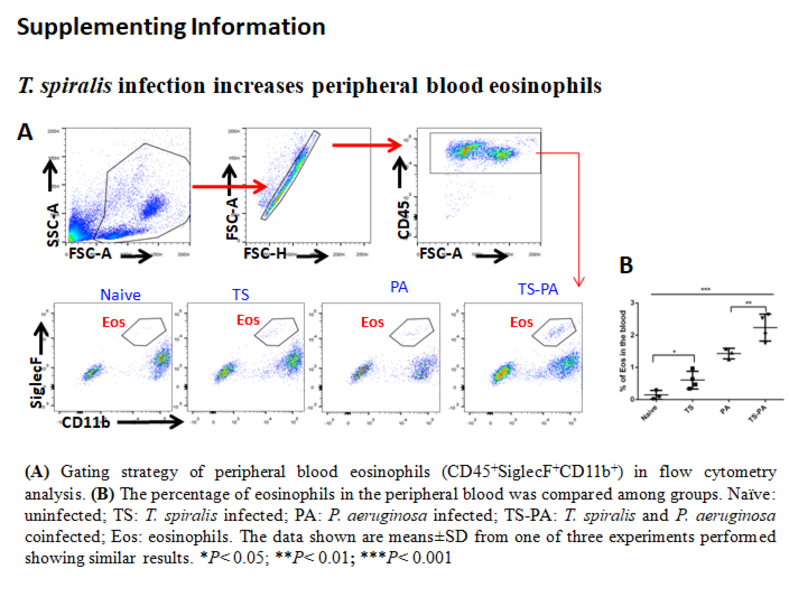

Supplement: S1 Fig — (A) Gating strategy of peripheral blood eosinophils (CD45+SiglecF+CD11b+) in flow cytometry analysis. (B) The percentage of eosinophils in the peripheral blood was compared among groups. Naïve: uninfected; TS: T. spiralis infected; PA: P. aeruginosa infected; TS-PA: T. spiralis and P. aeruginosa coinfected; Eos: eosinophils. The data shown are means±SD from one of three experiments performed showing similar results. *P<0.05; **P<0.01; ***P<0.001. (TIF) [file pntd.0010395.s002.tif]
